# Examining Hippocampal Mossy Fiber Synapses by 3D Electron Microscopy in Wildtype and Kirrel3 Knockout Mice

**DOI:** 10.1523/ENEURO.0088-17.2017

**Published:** 2017-06-19

**Authors:** E. Anne Martin, Derek Woodruff, Randi L. Rawson, Megan E. Williams

**Affiliations:** Department of Neurobiology and Anatomy, University of Utah School of Medicine, Salt Lake City, UT 84132

**Keywords:** electron microscopy, hippocampus, Kirrel3, mossy fiber, reconstruction, synapse

## Abstract

Neural circuits balance excitatory and inhibitory activity and disruptions in this balance are commonly found in neurodevelopmental disorders. Mice lacking the intellectual disability and autism-associated gene *Kirrel3* have an excitation-inhibition imbalance in the hippocampus but the precise synaptic changes underlying this functional defect are unknown. Kirrel3 is a homophilic adhesion molecule expressed in dentate gyrus (DG) and GABA neurons. It was suggested that the excitation-inhibition imbalance of hippocampal neurons in Kirrel3 knockout mice is due to loss of mossy fiber (MF) filopodia, which are DG axon protrusions thought to excite GABA neurons and thereby provide feed-forward inhibition to CA3 pyramidal neurons. Fewer filopodial structures were observed in Kirrel3 knockout mice but neither filopodial synapses nor DG en passant synapses, which also excite GABA neurons, were examined. Here, we used serial block-face scanning electron microscopy (SBEM) with 3D reconstruction to define the precise connectivity of MF filopodia and elucidate synaptic changes induced by Kirrel3 loss. Surprisingly, we discovered wildtype MF filopodia do not synapse exclusively onto GABA neurons as previously thought, but instead synapse with similar frequency onto GABA neurons and CA3 neurons. Moreover, Kirrel3 loss selectively reduces MF filopodial synapses onto GABA neurons but not those made onto CA3 neurons or en passant synapses. In sum, the selective loss of MF filopodial synapses with GABA neurons likely underlies the hippocampal activity imbalance observed in Kirrel3 knockout mice and may impact neural function in patients with Kirrel3-dependent neurodevelopmental disorders.

## Visual Abstract

**Figure:**
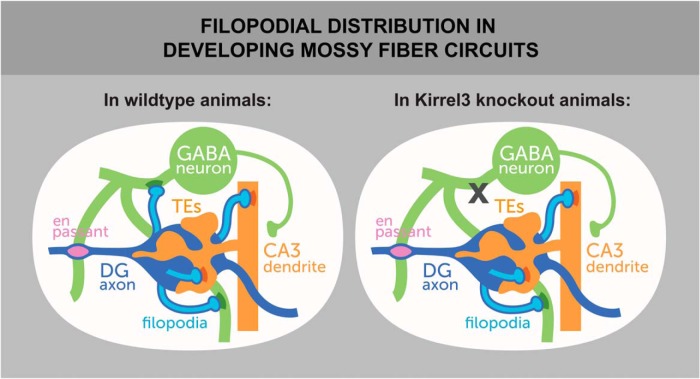

## Significance Statement

Point mutations and deletions in the gene *Kirrel3* are associated with neurodevelopmental disorders including autism, intellectual disability and Jacobsen’s syndrome, a chromosomal disorder that frequently includes epilepsy, autism, and intellectual disability. We studied the effect of losing Kirrel3 on synaptic connections in the mouse hippocampus, a brain region critical for learning and memory. We find that not only are a specific subset of synapses missing in Kirrel3 knockouts, but we also discovered a new synaptic connection within the hippocampus. The synaptic changes we found in mice lacking Kirrel3 shed new light on how a defective *Kirrel3* gene could cause neurodevelopmental disorders in humans.

## Introduction

**Figure 1.**
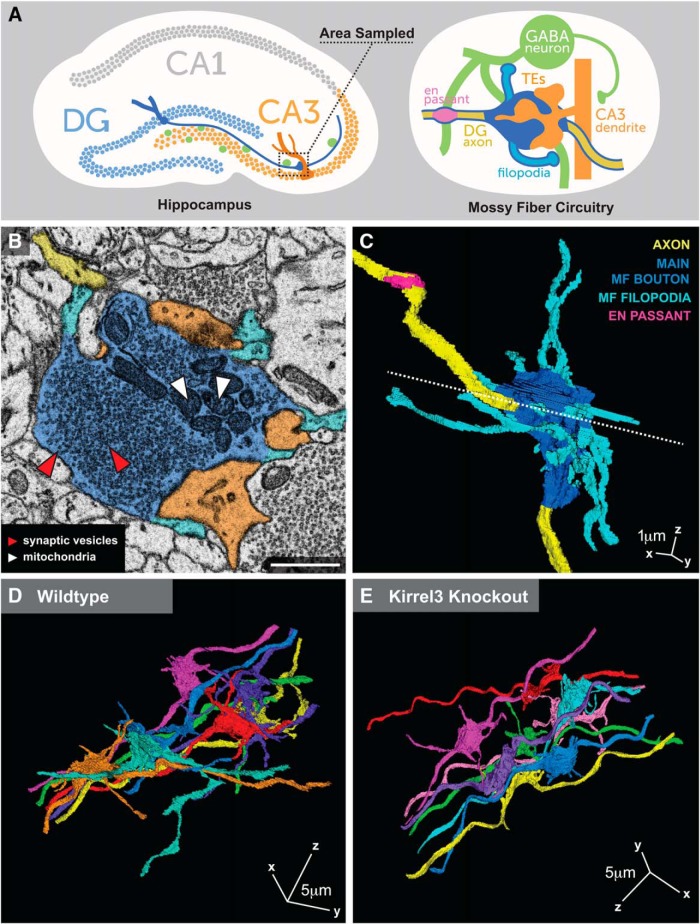
Reconstructing MF presynaptic complexes by SBEM. ***A***, Diagram of hippocampal MF circuitry. Boxed region notes the tissue area analyzed. On the right, the dark blue outline identifies the DG neuron. ***B***, Representative image of a wildtype SBEM section showing MF synapse components: main MF bouton (dark blue), MF filopodia (light blue), DG axon (yellow), TEs (orange), SVs (red arrows), and mitochondria (white arrows). Scale bar, 1 μm. ***C***, 3D reconstruction of the MF presynaptic complex in ***B***. The postsynaptic TEs are not shown. Dotted line in ***C*** shows the location of slice shown in ***B***. ***D***,***E***, Sample wildtype (***D***) and Kirrel3 knockout (***E***) 3D reconstructions showing eight MF presynaptic complexes each.

Mossy fiber (MF) presynaptic complexes are critical to hippocampal circuit function, especially for the mnemonic process of pattern separation (Rolls, 2013). Yet, the molecular mechanisms regulating their development are largely unknown. MF presynaptic complexes simultaneously connect glutamatergic DG neurons to glutamatergic CA3 pyramidal neurons (henceforth called “CA3 neurons”) and GABAergic interneurons (henceforth called “GABA neurons”; Fig. 1*A*). MF presynaptic complexes consist of a giant main bouton that is up to 100 times larger in volume than a typical presynapse (Shepherd and Harris, 1998; Wilke et al., 2013) and they synapse onto multiheaded CA3 spines called thorny excrescences (TEs; Fig. 1*A*). Because of its large size, numerous active zones, and proximity to the CA3 soma, release at a single main bouton profoundly impacts activity of the connected CA3 neuron (McBain, 2008). In addition, the MF presynaptic complex extends protrusions called MF filopodia to synapse with nearby GABA neurons (Fig. 1*A*; Amaral, 1979; Claiborne et al., 1986; Acsády et al., 1998). This MF filopodial synapse provides feed-forward inhibition to CA3 neurons (Frotscher, 1985; Deller et al., 1994; Bragin et al., 1995; Penttonen et al., 1997; Torborg et al., 2010). Distinct from the MF presynaptic complex, DG axons also activate GABA neurons via more typical en passant synapses (Fig. 1*A*). The diversity of DG presynaptic structures made with distinct postsynaptic partners suggests that different molecular mechanisms might be involved in establishing each type of synaptic connection, however, the molecules regulating MF synapse specificity remain largely unknown.

Kirrel3 is an immunoglobulin superfamily member that mediates homophilic cell adhesion (Gerke et al., 2005; Serizawa et al., 2006; Martin et al., 2015). Point mutations, copy number variations, and deletions in Kirrel3 have been repeatedly identified in patients with intellectual disability (Bhalla et al., 2008; Kaminsky et al., 2011; Talkowski et al., 2012), autism spectrum disorders (Ben-David and Shifman, 2012; Iossifov et al., 2012; Michaelson et al., 2012; Neale et al., 2012; Talkowski et al., 2012; Cheng et al., 2013; De Rubeis et al., 2014), and Jacobsen’s syndrome (Guerin et al., 2012). Given these associations with neurodevelopmental disorders affecting learning and memory, the role of Kirrel3 in the mouse hippocampus was recently investigated. Behaviorally, adult Kirrel3 knockout mice show a defective novel object recognition preference, as well as hyperactivity in a familiar environment (Choi et al., 2015). Molecularly, Kirrel3 is only expressed in DG neurons and a subset of GABA neurons in the hippocampus, yet functionally, Kirrel3 knockout mice have an increase in DG and CA3 neuron activity compared with wildtype mice (Martin et al., 2015; Roh et al., 2017). These functional defects could be explained by the morphologic observation that Kirrel3 knockout mice have fewer MF filopodia than wildtype mice (Martin et al., 2015), which may decrease feed-forward inhibition to CA3 neurons. These prior studies support the hypothesis that Kirrel3 acts via its homophilic extracellular domain to regulate synapse formation between DG MF filopodia and GABA neurons. However, hippocampal synapses in Kirrel3 knockout mice have not yet been directly examined.

Here, we define DG presynapse connectivity in wildtype and Kirrel3 knockout mice using serial block-face scanning electron microscopy (SBEM). Though several 3D electron microscopy (EM) studies of wildtype MF synapses exist (Rollenhagen et al., 2007; Faulkner et al., 2008; Wilke et al., 2013), none assessed MF filopodia or en passant synapse connectivity. Thus, our study not only provides a high-resolution analysis of hippocampal synaptic defects in Kirrel3 knockout mice but also provides the first precise description of MF filopodia and en passant connectivity in wildtype mice.

## Materials and Methods

### Animals

Tissue from two different sets of wildtype and knockout littermate mice were imaged to create four independent SBEM datasets. P14 littermates consisting of a Kirrel3 wildtype (female) and a Kirrel3 knockout (female) were used for datasets 1 and 2 and P14 littermates from a different breeding pair consisting of a Kirrel3 wildtype (male) and a Kirrel3 knockout (female) were used for datasets 3 and 4. All animals and experiments were maintained and conducted in accordance with the NIH guidelines on the care and use of animals and approved by the University of Utah School of Medicine Institutional Animal Care and Use Committee. Generation of Kirrel3 knockout mice was previously described (Prince et al., 2013).

### Tissue fixation and processing

Mice were anesthetized and perfused at a rate of 6 ml/min with 2.5% glutaraldehyde and 4% paraformaldehyde in 0.1 M sodium cacodylate buffer. The brains were removed and stored overnight at 4°C in perfusion solution. The next day the brains were sliced in cold PBS into 200-μm coronal sections and sent to Renovo Neural (RRID: SCR_001035) for processing and imaging on a Zeiss Sigma VP Scanning Electron Microscope with a Gatan 3View door.

### Image stacks

Each dataset contains 400-500 serial images. In datasets 1 (wildtype) and 2 (knockout), images are 45 × 45 μm at 7 nm/pixel resolution with a z-depth of 70 nm to cover a volume of 56,700 μm^3^. In datasets 3 (wildtype) and 4 (knockout), images are 37 × 37 μm at 6 nm/pixel resolution with a z-depth of 70 nm to cover a volume of 47,915 μm^3^. Beam penetration for each was 25–30 nm. TrakEM2 software was used to assemble images for 3D reconstruction and analysis (Cardona et al., 2012; RRID: SCR_008954). All reconstruction and analysis was done blind to genotype. MF presynaptic complexes near the center of the dataset were randomly selected for reconstruction. MF presynaptic complexes with MF filopodia extending out of the dataset were not analyzed.

### Determination of structures

Main MF boutons were identified as an axon enlargement filled with synaptic vesicles (SVs) adjacent to postsynaptic densities (PSDs) on CA3 TEs. MF filopodia were identified as a projection extending at least 0.5 μm off of the main MF bouton with a clear neck structure lacking SVs from the main MF bouton. SV clusters are defined as a group of 10 or more SVs. Synapse-free MF filopodia contained no SV clusters. Partial MF filopodial synapses are defined as SV clusters with no associated PSD. Complete MF filopodial synapses are defined as an SV cluster adjacent to a PSD. Complete and partial synapses could be located anywhere along the length of the MF filopodia. En passant synapses were identified by locating a main MF bouton and following its axon through the dataset. En passant synapses are defined as an SV cluster located in the DG axon with an adjacent PSD.

### Dendrite classification

Synapses were classified as being made onto a CA3 neuron, GABA neuron, or unknown. GABA dendrites were identified as either aspiny or had simple protrusions that were not CA3 TEs. CA3 pyramidal neurons were identified by the presence of TEs (multiheaded spines that synapse with large main MF boutons. Some synapses were marked as having unknown partners because they were onto dendritic elements that could not be followed through the dataset.

### Image measurements, statistics, and image presentation

Volumetric measurements were generated in TrakEM2 software. Statistics were calculated in Prism (GraphPad, RRID: SCR_002798). In all cases, data were tested for normality using the D'Agostino and Pearson test. If all datasets were normal, parametric tests were used to compare genotypes. Otherwise, nonparametric tests were used. For publication, EM images were adjusted for visibility using Photoshop, but the entire field of view and all images were always adjusted in the same manner. All analysis and quantification was done using raw image data.

## Results

### Reconstructing MF presynaptic complexes by SBEM

Because MF presynaptic complexes are giant structures with elaborate features, complete analysis of their ultrastructure requires 3D EM. SBEM excellently suits this task as it generates hundreds of well-aligned serial EM images at nanoscale resolution (Denk and Horstmann, 2004). Given that Kirrel3 is highly expressed in DG neurons and is associated with developmental disorders, we analyzed DG presynapses by EM during development at age postnatal day 14 (P14). We collected four SBEM datasets, each covering at least 47,915 μm^3^ of tissue from two P14 wildtype and two P14 Kirrel3 knockout mice. Because Kirrel3 is selectively expressed in DG and GABA neurons and mediates homophilic adhesion, it suggests Kirrel3 will regulate DG-GABA synapses. Thus, in all datasets we sampled the stratum lucidum of the dorsal hippocampus, which contains DG axons, GABA neurons, and CA3 neurons (Fig. 1*A*).

TrakEM2 software (Cardona et al., 2012) was used to manually reconstruct synapses. We reconstructed 15 MF presynaptic complexes in each wildtype volume, 19 in one Kirrel3 knockout volume, and 15 in the other knockout volume for a total of 30 wildtype and 34 knockout MF complexes and all associated MF filopodia. All tracing and analysis was done blind to genotype to avoid selection biases. Main MF boutons were unambiguously identified by their large size, high density of SVs, and scattered mitochondria (Fig. 1*B*). After selection of a main MF bouton, all attached filopodia and the axon were fully traced and reconstructed in 3D (Fig. 1*B*,*C*; Movie 1). Representative images of eight reconstructed MF complexes from a wildtype mouse and a Kirrel3 knockout mouse are shown (Fig. 1*D*,*E*).

### MF filopodia exist in three states during development

**Figure 2.**
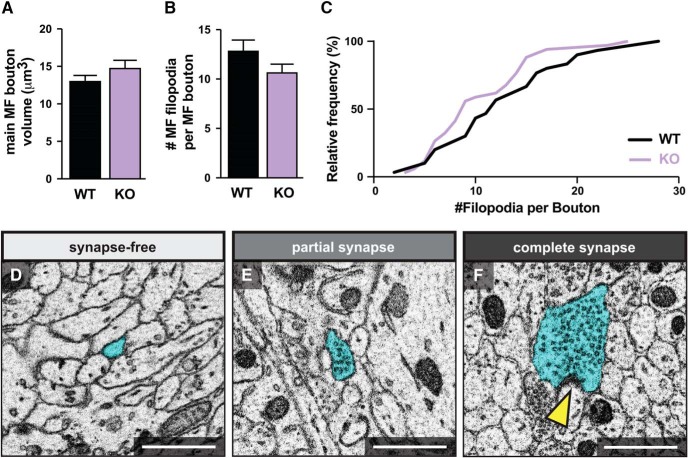
MF filopodia exist in three states during development. ***A***, The average volume of the main MF bouton per genotype. *p* = 0.5102. ***B***, The average number of MF filopodia per main MF bouton. *p* = 0.1323. ***C***, Cumulative histogram showing the number of MF filopodia per main MF bouton in each genotype. Note: Kirrel3 knockouts tend to have MF boutons with fewer filopodia. *p* = 0.2360. ***D–F***, Representative images of MF filopodia in each of the three synaptic states (synapse-free, partial, complete). Yellow arrow indicates PSD. For all graphs: sample size: WT = 30 and KO = 34 main MF boutons. Error bars show mean ± SEM. Scale bars, 1 μm.

After reconstructing at least 30 MF presynaptic complexes from each genotype, we find that loss of Kirrel3 does not cause statistically significant differences in the volume of the main MF bouton (Fig. 2*A*) or the mean number of MF filopodia per main MF bouton (Fig. 2*B*) compared with wildtype mice. However, consistent with a previous report (Martin et al., 2015), a cumulative histogram of the data reveals that MF presynaptic complexes from Kirrel3 knockout mice tend to have fewer MF filopodia per main MF bouton (Fig. 2*C*).

It is currently unknown if MF filopodia density directly correlates with synapse density, which is ultimately the most critical factor impacting MF circuit function. Therefore, we next analyzed the density and postsynaptic targets of synapses housed in MF filopodia. We identified three states with which to classify MF filopodia. First, some MF filopodia contain no SV clusters nor are they adjacent to any PSDs anywhere along their length (Fig. 2*D*). We refer to these MF filopodia as “synapse free.” Second, some MF filopodia contain clusters of 10 or more SVs but no visible corresponding PSD (Fig. 2*E*). Presynaptic elements are commonly found before postsynaptic elements are present (Amaral and Dent, 1981; Friedman et al., 2000; Wilke et al., 2013), and we postulate that these SV clusters either mark future synaptic sites or have PSDs that are not visible due to plane of sectioning. We refer to these as “partial synapses.” Third, some MF filopodia contain bona fide synapses with SV clusters adjacent to a PSD (Fig. 2*F*). We refer to these as “complete synapses.”

### Kirrel3 regulates formation of MF filopodial synapses

**Figure 3.**
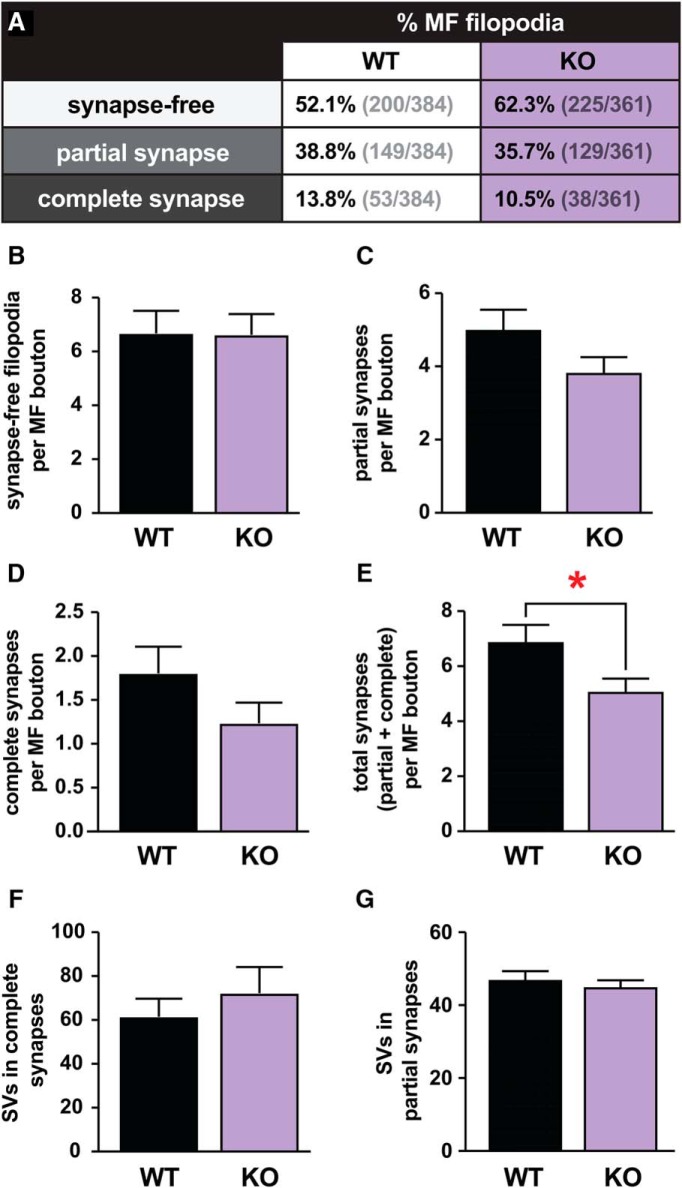
Kirrel3 regulates MF filopodial synapse density. ***A***, Table showing percentage of MF filopodia that are synapse-free, or contain partial or complete synapses. Note: An individual MF filopodia can have multiple synapses, thus the totals do not sum to 100%. ***B–D***, Quantification of the number of synapse-free MF filopodia (***B***), partial synapses (***C***), and complete synapses (***D***) per main MF bouton by genotype. ***E***, Quantification of total number of synapses (partial + complete) per main MF bouton. *p = 0.0357 using a two-tailed *t* test. Sample size for ***A–E***: WT = 30 and KO = 34 main MF boutons. ***F***, Quantification of the number of SVs in complete synapses. Sample size for ***F***: WT = 55 and KO = 42 synapses. ***G***, Quantification of the number of SVs in partial synapses. Sample size for ***G***: WT = 150 and KO = 131 synapses. For all graphs: Unless noted, *p* > 0.05 (Table 1). Error bars show mean ± SEM.

When comparing the total percentage of MF filopodia in each synaptic state by genotype, we find Kirrel3 knockout mice have a greater percentage of MF filopodia that are synapse-free and a lower percentage that contain partial and complete synapses compared with wildtype (Fig. 3*A*). Next, we analyzed the average number of MF filopodia that exist in each state per main MF bouton, which comprises the key functional unit for a DG neuron. When analyzed this way, the average number of synapse-free MF filopodia per main MF bouton is similar between wildtype and knockout (Fig. 3*B*). This suggests that the missing MF filopodia (Fig. 2*B*,*C*) are those that would normally contain a synapse. In support of this, we observe reductions in both synapse types per main bouton in Kirrel3 knockout mice (Fig. 3*C*,*D*), and when partial and complete synapses are combined, Kirrel3 knockout mice have significantly fewer synapses per main MF bouton than wildtype mice (Fig. 3*E*). The average number of SVs in synapses was similar between the genotypes (Fig. 3*F*,*G*). Thus, our data indicate that, compared with wildtype mice, Kirrel3 knockout mice have fewer MF filopodia with synapses, but the synapses that do form have normal ultrastructure.[Table T1]

**Table 1. T1:** Statistics

Graph	Data structure	Type of test	*p* value
Figure 2*A*	Nonparametric	Mann-Whitney test	0.5102
Figure 2*B*	Normal distribution	Unpaired *t* test	0.1323
Figure 2*C*	Normal distribution	Kolmogorov-Smirnov test	0.2360
Figure 3*B*	Normal distribution	Unpaired *t* test	0.9781
Figure 3*C*	Normal distribution	Unpaired *t* test	0.1140
Figure 3*D*	Nonparametric	Mann-Whitney test	0.2061
Figure 3*E*	Normal distribution	Unpaired *t* test	0.0357
Figure 3*F*	Nonparametric	Mann-Whitney test	0.2595
Figure 3*G*	Nonparametric	Mann-Whitney test	0.8720
Figure 4*J*	Nonparametric	Mann-Whitney test	0.0075
Figure 4*K*	Nonparametric	Mann-Whitney test	0.7906
Figure 4*M*	Nonparametric	Kruskal-Wallis test	0.0051
Figure 4*N*	Nonparametric	Kruskal-Wallis test	0.0153
Figure 4*O*	Nonparametric	Kruskal-Wallis test	0.0973
Figure 4*P*	Nonparametric	Kruskal-Wallis test	0.1268
Figure 6*E*	Nonparametric	Mann-Whitney test	0.5208

### Kirrel3 selectively regulates MF filopodial synapses onto GABA neurons

**Figure 4.**
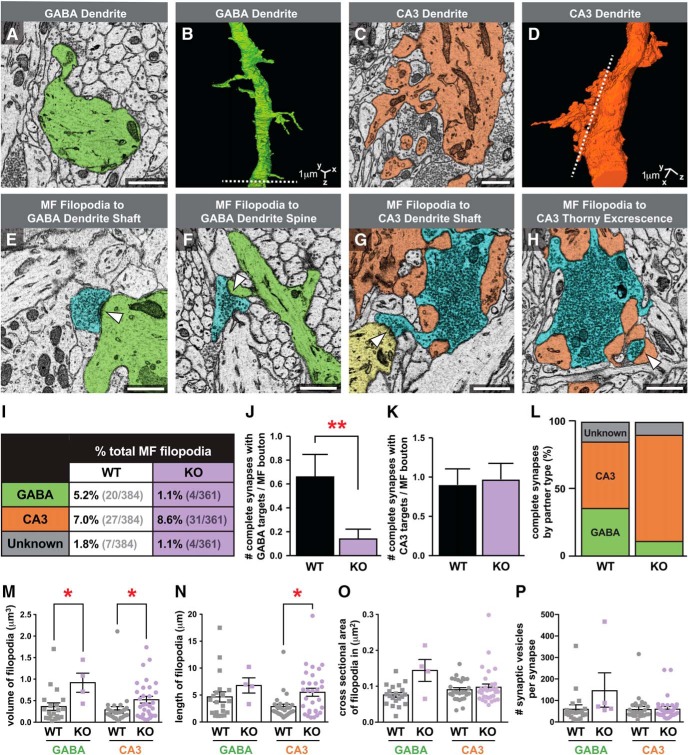
Kirrel3 regulates MF filopodial synapses with GABA neurons, but not CA3 neurons. ***A***,***B***, 2D (***A***) and 3D (***B***) example of a GABA dendrite (green) with simple spine-like protrusions and no PSDs with main MF boutons. Dotted line in ***B*** shows the location of slice shown in ***A***. ***C***,***D***, 2D (***C***) and 3D (***D***) example of a CA3 neuron (orange) with multiheaded TE spines and many PSDs with main MF boutons. Dotted line in ***D*** shows the location of slice shown in ***C***. ***E***,***F***, Representative images of complete MF filopodial synapses made onto a GABA dendritic shaft (***E***) and a GABA spine (***F***). MF filopodia (blue), GABA dendrite (green), synapses indicated by white arrows. ***G***,***H***, Representative images of complete MF filopodial synapses made onto a second CA3 dendritic shaft (***G***) and a CA3 TE (***H***). Main MF bouton and MF filopodia (blue), CA3 dendrite and TEs (orange), second CA3 dendrite (yellow), synapses indicated by white arrows. ***I–L***, Quantification of MF filopodial synapses onto different types of postsynaptic neurons. ***I***, Percentage of wildtype or Kirrel3 knockout MF filopodia making complete synapses with indicated postsynaptic partner type. ***J***, Number of complete MF filopodial synapses onto GABA neurons per main MF bouton. **p = 0.0075 by Mann-Whitney test. ***K***, Number of complete MF filopodial synapses onto CA3 neurons per main MF bouton. ***L***, Including only the MF filopodia that make a complete synapse, the percentage of each partner type is shown. Sample size for ***I–L***: WT = 30 and KO = 34 main MF boutons. Unless indicated *p* > 0.05. ***M–P***, Comparison of the morphology of filopodia synapsing with GABA versus CA3 neurons in wildtype and Kirrel3 knockout mice using the Kruskal-Wallis test with multiple comparisons. ***M***, Volume of filopodia in μm^3^. GABA WT to KO *p = 0.0419, CA3 WT to KO *p = 0.0507. ***N***, Length of filopodia in micrometers. *p = 0.0299. ***O***, Cross sectional area in square micrometers. *p* > 0.05. ***P***, Number of SVs per synapse. *p* > 0.05. Sample size for ***M–O***: WT GABA = 20, KO GABA = 4, WT CA3 = 27, KO CA3 = 31. Sample size for ***P***: WT GABA = 20, KO GABA = 5, WT CA3 = 27, KO CA3 = 33. For entire figure, error bars show mean ± SEM. Scale bars, 1 μm.

Next we determined the postsynaptic targets of MF filopodial synapses. Broadly defined there are two types of potential postsynaptic target neurons in the stratum lucidum; GABA neurons and CA3 neurons. The two target cell types can be readily identified by their dendritic morphology. GABA neurons can be aspiny or spiny but their dendrites always lack multiheaded TE spines (Fig. 4*A*,*B*). Conversely, CA3 dendrites always have multiheaded TE spines making multiple synapses with main MF boutons (Fig. 4*C*,*D*).

It is widely accepted that MF filopodia only synapse onto GABA neurons (Amaral, 1979; Claiborne et al., 1986; Acsády et al., 1998). Therefore, we were surprised to observe that, in P14 wildtype mice, MF filopodia form complete synapses with similar frequency onto GABA dendrites (Fig. 4*E*,*F*,*I*) as CA3 dendrites (Fig. 4*G-I*). Most GABA dendrites contacted by MF filopodia have simple spiny protrusions as previously observed (Acsády et al., 1998), and synaptic contacts are found on both GABA dendritic shafts (Fig. 4*E*) and spiny protrusions (Fig. 4*F*). Similarly, when MF filopodia synapse onto CA3 neurons, we observed synaptic contacts on CA3 dendritic shafts (Fig. 4*G*) and TE spines (Fig. 4*H*).

MF filopodia also synapse onto both GABA and CA3 neurons in Kirrel3 knockout mice. However, knockout mice have a clear shift in the distribution of postsynaptic partners. Compared with wildtype mice, Kirrel3 knockout mice develop significantly fewer complete synapses with GABA neurons (Fig. 4*J*). In contrast, the number of complete MF filopodial synapses made onto CA3 neurons is similar between wildtype and Kirrel3 knockout mice (Fig. 4*K*). The net effect of these changes is a dramatic decrease in the ratio of MF filopodial synapses made onto GABA versus CA3 neurons in mice lacking Kirrel3 compared with wildtype (Fig. 4*L*).

We then examined if filopodia synapsing with GABA neurons have a different morphological signature from filopodia synapsing with CA3 neurons, and if these characteristics change upon Kirrel3 loss (Fig. 4*M–P*). For this analysis, we only included only filopodia that have a complete synapse onto an identified GABA or CA3 neuron. Because Kirrel3 knockout mice have significantly fewer filopodia-GABA synapses than wildtype mice, the sample size for this category was small (*n* = 4 filopodia with five complete synapses). Nonetheless, this new analysis indicates that, on average, filopodia synapsing onto either GABA or CA3 neurons have a similar morphology and SV composition in wildtype mice (Fig. 4*O*,*P*). However, we did observe some differences in filopodia morphology between wildtype and Kirrel3 knockout mice. In particular, the total volume of both classes of filopodia (those connected to either GABA or CA3 neurons) is increased on Kirrel3 loss (Fig. 4*M*). For filopodia synapsing onto CA3 neurons, the increased volume is clearly due to increased length (Fig. 4*N*) and not an increase in cross-sectional area (Fig. 4*O*). For filopodia synapsing onto GABA neurons, the increased volume may be due to a combination of increased length and cross-sectional area but neither parameter is significantly different (Fig. 4*N*,*O*) and we hesitate to draw strong conclusions based on the low sample size of filopodia synapsing with GABA neurons in Kirrel3 knockout mice.

### En passant connectivity is unperturbed by Kirrel3 loss

**Figure 5.**
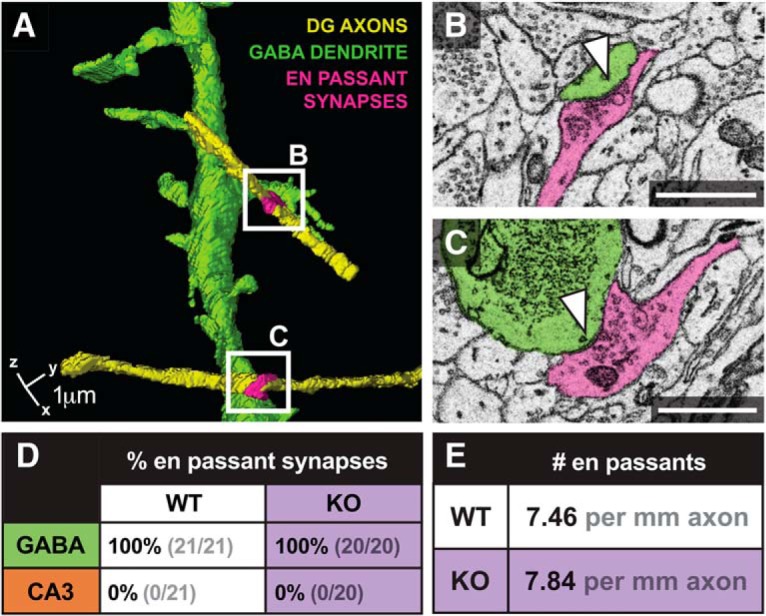
Kirrel3 does not regulate en passant synapse density. ***A–C***, 3D example (***A***) of a spiny GABA dendrite targeted by en passant synapses from two separate DG axons. Synapses magnified in ***B***, ***C***. GABA dendrite (green), DG axon (yellow), en passant synapse (pink). ***D***, Percentage of en passant synapses made onto GABA and CA3 dendrites from 73 WT and 68 KO DG axon segments. ***E***, Table reporting the number of en passant synapses per millimeter axon. Scale bars, 1 μm.

Next, we analyzed formation of DG en passant synapses in wildtype and Kirrel3 knockout mice. DG en passant synapses are readily identifiable by EM as clusters of SVs in the axon shaft adjacent to a PSD (Fig. 5*A-C*). Like MF filopodia, en passant synapses are thought to synapse exclusively onto GABA neurons (Acsády et al., 1998). Given that Kirrel3 is expressed specifically by DG and GABA neurons, we reasoned that in addition to regulating DG MF filopodia to GABA synapses, Kirrel3 may also regulate formation of DG en passant to GABA synapses. Consistent with the existing literature, 100% of DG en passant synapses observed in our datasets synapse with GABA neurons (Fig. 5*D*). However, interestingly, the loss of Kirrel3 has no effect on the density of en passant synapses in DG axons (Fig. 5*E*). This indicates Kirrel3 has a highly specific function regulating DG MF filopodia to GABA neuron synapses but not DG axon to GABA neuron synapses.

### Other morphologic features of MF filopodia

**Figure 6.**
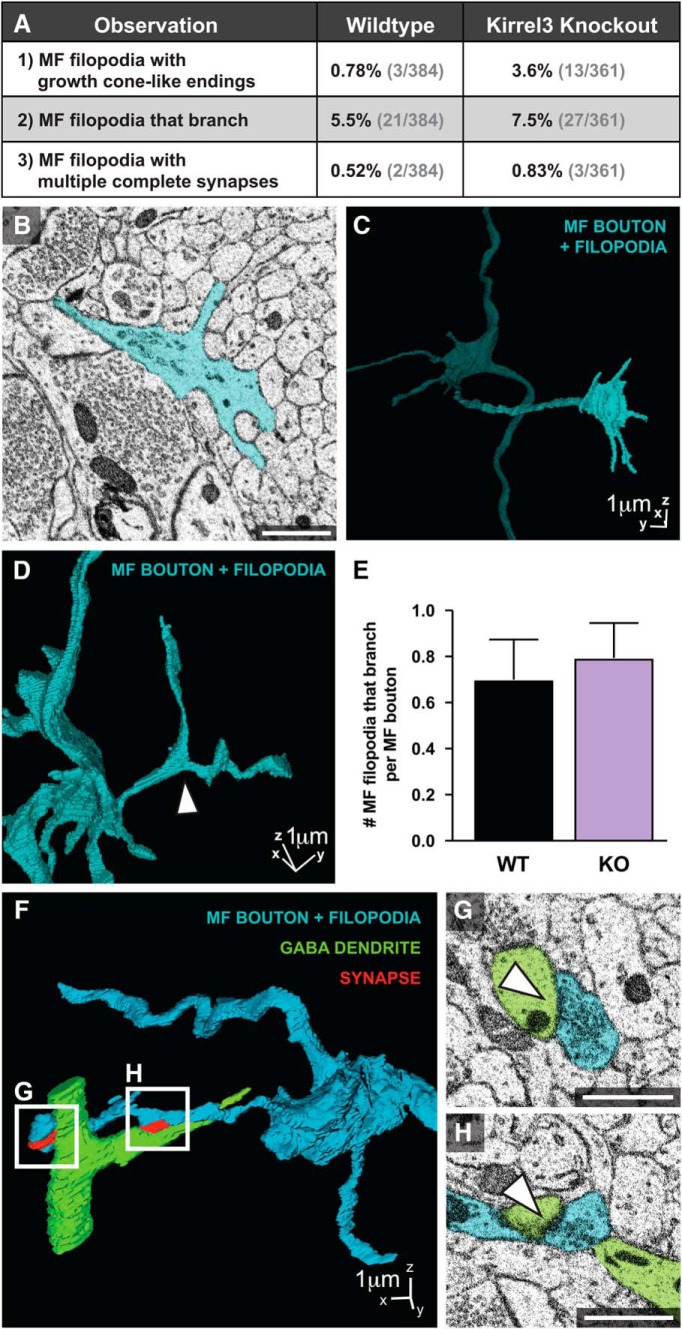
Additional morphologic features of MF circuits. ***A***, Table reporting the percentage of wildtype and Kirrel3 knockout MF filopodia with indicated features. ***B***, ***C***, Representative 2D (***B***) and 3D (***C***) example of a MF filopodia ending in a growth cone-like structure. ***D***, 3D example of a branched MF filopodia. White arrow indicates branch point. ***E***, Quantification of the number of branching MF filopodia per main MF bouton. Sample size: WT = 30 and KO = 34 main MF boutons. *p* > 0.05. Error bars show mean ± SEM. ***F–H***. 3D example of one MF filopodia making multiple synapses onto one GABA neuron (***F***), synapses magnified in ***G***, ***H*** shown in 2D. Main MF bouton with associated MF filopodia (blue), GABA dendrite (green), synapses (red). Scale bars, 1 μm.

Given that this is the first in-depth 3D analysis of MF filopodia connectivity, we noted the presence of several unusual MF filopodia structures. First, we occasionally observed growth cone-like endings at the tips of MF filopodia (Fig. 6*A-C*). These structures had no synapses and few, if any, SVs along their length. Given that our data are from P14 mice that are near the peak of DG synaptogenesis, they likely represent newly formed MF filopodia in search of a target neuron. Second, we observe branched MF filopodia (Fig. 6*A*,*D*,*E*). We sought to determine if MF filopodia from Kirrel3 knockout mice have fewer branches because an ortholog of Kirrel3, SYG-1, regulates axonal branch generation in *Caenorhabditis elegans* motor neurons (Chia et al., 2014). However, we find little difference in filopodia branching between wildtype and Kirrel3 knockout mice (Fig. 6*E*). Third, though it is often assumed that MF filopodia contain only one terminal synapse at the MF filopodia tip, we observe that individual MF filopodia can house multiple synapses with some located in the MF filopodia shaft (Fig. 6*A*,*F-H*). It will be interesting to determine in a future study whether these features of growth cone-like ends, branches, and multiple synapses are developmental in origin or are dynamically maintained in adult mice to allow circuit flexibility.

## Discussion

Here, we present a rigorous ultrastructural analysis of MF filopodial connectivity in the developing mammalian brain in wildtype and Kirrel3 knockout mice. First, we discovered contrary to previous reports, MF filopodia synapse with similar frequency onto GABA and CA3 neurons. Second, we show Kirrel3 is selectively required for normal synapse density between DG MF filopodia and GABA neurons but not CA3 neurons. Third, we discovered Kirrel3 does not regulate DG-GABA en passant synapse formation.

To our knowledge, this is the first report of MF filopodia synapsing with CA3 pyramidal neurons. Previous work indicated that MF filopodia only synapse onto GABA neurons (Acsády et al., 1998; McBain, 2008; Rollenhagen, 2010). However, prior work examined MF filopodia connectivity at an unspecified age without the use of current, high-throughput EM technologies (Acsády et al., 1998). In contrast, our study was done on juvenile P14 animals and we analyzed >350 MF filopodia per genotype, providing an improved sample size. Thus, it is possible MF filopodia-CA3 synapses exist in the adult and were previously missed due to factors such as sample size or imaging method. It is also possible MF filopodia-CA3 synapses are a developmental phenomenon that are selectively pruned by adulthood. Future studies on adult mice are needed to distinguish between these two possibilities. In addition, we observed that filopodia synapsing onto either GABA or CA3 neurons have a similar morphology and SV composition in wildtype mice, but those synapsing onto CA3 neurons in Kirrel3 knockout mice are significantly longer. Because Kirrel3 knockout mice have so few filopodia synapsing with GABA neurons, we postulate the increased length of filopodia synapsing with CA3 neurons could be due to the extra availability of resources, increased drive to search for a postsynaptic partner, or delayed maturity.

Why might the MF circuit benefit from having MF filopodia synapse onto both GABA and CA3 neurons? MF filopodia are highly plastic, motile structures (Tashiro et al., 2003) and are likely better suited to quickly adapt to changing circuit needs than the large anchored main MF bouton. Over time, circuits adapt to maintain a homeostatic balance of excitatory and inhibitory activity (Turrigiano, 2012; Nelson and Valakh, 2015) and having flexible MF filopodia that can increase or decrease input to GABA versus CA3 neurons may be one way the hippocampus maintains this balance. It was shown that MF presynaptic complexes and MF filopodia increase in response to enriched environment and learning tasks such as fear conditioning and the Morris water maze (Bednarek and Caroni, 2011; Ruediger et al., 2011). However, it is not known if and where these newly added MF filopodia make synapses. Knowing whether these newly added adult MF filopodia preferentially synapse onto GABA neurons or CA3 neurons would provide insight toward understanding hippocampal circuit changes underlying learning and memory.

In addition to more precisely defining MF connectivity in wildtype mice, our study suggests Kirrel3 is a highly selective target-specificity molecule. We show Kirrel3 is specifically required to establish the appropriate density of MF filopodia-GABA synapses, but not MF filopodia-CA3 synapses or DG en passant-GABA synapses during development. It is possible that loss of Kirrel3 causes a permanent reduction in filopodia-GABA synapses or, alternatively, the loss of Kirrel3 may cause a delayed maturation of filopodia-GABA synapses. The current study conducted at one developmental time point cannot distinguish between these possibilities but future work analyzing filopodial synapse development over time can address this mechanism. Nonetheless, Kirrel3 is a homophilic, transmembrane molecule selectively expressed in DG and GABA neurons (Martin et al., 2015). Thus, our results support the hypothesis that trans-cellular homophilic Kirrel3 binding selectively stabilizes MF filopodial contacts between DG axons and GABA dendrites. Kirrel3 may also provide a pre- and postsynaptic signal to actively induce synapse formation via its intracellular domain. In support, mammalian Kirrel3 was shown to interact with the synaptic molecules CASK (Gerke et al., 2006; Bhalla et al., 2008), PICK1 (Höhne et al., 2011), and recently PSD95 (Roh et al., 2017), but details of how Kirrel3 signals *in vivo* remain unknown.

It is noteworthy that not all MF filopodia-GABA synapses are eliminated in Kirrel3 knockout mice. Kirrel3 is widely expressed by DG neurons but is expressed only by a subpopulation of calbindin-positive GABA neurons. These Kirrel3-positive interneurons make up just 19% of all GABA neurons present in area CA3 (Martin et al., 2015). Thus Kirrel3 knockout mice may selectively lack all input to this subpopulation of GABA neurons and the MF filopodia-GABA synapses that remain may connect DG neurons to Kirrel3-negative GABAergic neurons via alternative mechanisms. Additionally, we observed no change in en passant synapse number on loss of Kirrel3. It is possible Kirrel3 does not localize along the DG axon or that en passant synapses selectively target Kirrel3-negative GABA neurons. The latter possibility would suggest DG neurons use MF filopodial and en passant synapses to differentially communicate with distinct types of GABA neurons.

In conclusion, our results indicate Kirrel3 is required for formation of a subset of hippocampal DG-GABA synapses. Loss of DG-GABA synapses is expected to reduce feed-forward inhibition to CA3 neurons and likely explains the prior observation that juvenile Kirrel3 knockout mice have over-active CA3 neurons (Martin et al., 2015). Selective loss of DG-GABA synapses may contribute to the etiology of Kirrel3-dependent neurodevelopmental disorders by causing an excitation/inhibition imbalance in hippocampal circuits and a better understanding of the precise synaptic defects caused by loss of Kirrel3 may ultimately lead to more effective treatments.

Movie 1. >MF presynaptic complex shown in Figure 1*B*,*C*. 10.1523/ENEURO.0088-17.2017.video.1

## Synthesis

Reviewing Editor: Vivian Budnik, University of Massachusetts Decisions are customarily a result of the Reviewing Editor and the peer reviewers coming together and discussing their recommendations until a consensus is reached. When revisions are invited, a fact-based synthesis statement explaining their decision and outlining what is needed to prepare a revision will be listed below. The following reviewer(s) agreed to reveal their identity: Elva Diaz

summary of discussion

Both reviewers praised the manuscript for the high quality of the data. However, both reviewers also believed that additional work should be conducted to make this paper suitable for publication in eneuro. In addition, the authors should address the concerns outlined below.

Experimental

1. Given the observation that MF filopodia project to CA3 pyramidal neurons, the authors should perform a thorough structural analysis to determine if there are structural differences between filopodia projecting either onto GABA or CA3 neurons. In particular, the dimension (length, width and volume) of filopodia and SV density should be compared.

Data analysis

2. The p-values for all of the graphs in Figure 2 should be stated in the legend.

Results

3. When analyzing the complexity of filopodia by Sholl analysis, the difference of MF morphology between WT and Kirrel KO mice should be noted.

4. Define “TE spines” within the text when first used.

5. The sub-heading “MF filopodia exist in three synaptic states during development” is somewhat confusing because the first 'synaptic state' is 'synapse-free' which is not a 'synaptic state.' Simply referring to 'three states' would be better.

Title and Discussion

6. The main conclusions of the manuscript should be tempered, because an alternative interpretation of results is that synapses between the dentate gyrus mossy fiber filopodia and GABAergic interneurons might be delayed in the mutant. This should be discussed.

7. As authors are not addressing mechanisms in this paper, it would be desirable to change the word “regulates” in the title.

Figures

8. The region in Figure 1C should be indicated in Figure 1B. A suggestion is to match the colors in Figure 1A with the colors used in Figure 1B-C.

9. What area is Figure 4A and Figure 4C derived from in Figure 4B and Figure 4D, respectively?
